# Long Non-Coding RNA BST2/BISPR is Induced by IFN and Regulates the Expression of the Antiviral Factor Tetherin

**DOI:** 10.3389/fimmu.2014.00655

**Published:** 2015-01-09

**Authors:** Marina Barriocanal, Elena Carnero, Victor Segura, Puri Fortes

**Affiliations:** ^1^Department of Gene Therapy and Hepatology, Center for Applied Medical Research (CIMA), University of Navarra, Pamplona, Spain; ^2^Bioinformatics Unit, Center for Applied Medical Research (CIMA), University of Navarra, Pamplona, Spain

**Keywords:** IFN, lncRNAs, BST2, ISG15, HCV, tetherin

## Abstract

Many long non-coding RNAs (lncRNAs) are expressed in cells but only a few have been well characterized. In these cases, lncRNAs have been shown to be key regulators of several cellular processes. Therefore, there is a great need to understand the function of more lncRNAs and their regulation in response to stimuli. Interferon (IFN) is a key molecule in the cellular antiviral response. IFN binding to its receptor activates transcription of several IFN-stimulated genes (ISGs) that function as potent antivirals. In addition, several ISGs are positive or negative regulators of the IFN pathway. This is essential to ensure a strong antiviral response and a later return of the cell to homeostasis. As the ISGs described to date are coding genes, we sought to determine whether IFN also regulates the expression of long non-coding ISGs. To this aim, we used RNA sequencing to analyze the transcriptome of control and HuH7 cells treated with IFNα2. The results show that IFN-treatment regulates the expression of several unknown non-coding transcripts. We have validated two lncRNAs upregulated after treatment with different doses of type I IFNα2 in different cells or with type III IFNλ. These lncRNAs were also induced by influenza and vesicular stomatitis virus mutants unable to block the IFN response, but not by several wild-type lytic viruses tested. These lncRNA genes were named lncISG15 and lncBST2 as they are located close to ISGs ISG15 and BST2, respectively. Interestingly, inhibition experiments showed that lncBST2 is a positive regulator of BST2. Therefore lncBST2 has been renamed BISPR, from BST2 IFN-stimulated positive regulator. Our results may have therapeutic implications as lncBST2/BISPR, but also lncISG15 and their coding neighbors, are increased in cells infected with hepatitis C virus and in the liver of infected patients. These results allow us to hypothesize that several lncRNAs could be activated by IFN to control the potency of the antiviral IFN response.

## Introduction

The interferon (IFN)-mediated innate immune response provides a potent defense against pathogens (1). Upon invasion, pathogen-associated molecular patterns (PAMPs) are detected by specific receptors in the cells. These can be located on the surface of the cell, as in the case of Toll-like receptors (TLRs), or intracellularly, as in the case of the retinoic acid-inducible gene I (RIG-I). PAMP recognition triggers a series of signaling cascades that lead to the production and secretion of Type I IFN. Type I IFN (IFNα, IFNβ, and others) binds to IFN receptors present on the surface of all cell types and activates Janus-activated kinase/signal transducer and activator of transcription (JAK/STAT) signaling. This gives rise to the nuclear translocation of the STAT1/STAT2/IRF9 (IFN regulatory factor 9) complex that binds IFN-stimulated response elements (ISRE) in the promoters of IFN-stimulated genes (ISGs) and activates their transcription. A similar response is induced by Type III IFN (IFNλ) upon binding to its receptor (2, 3). In contrast, Type II IFN or IFNγ, produced by cells of the immune system, binds to the widely expressed IFNγ receptor (4, 5) leading to nuclear translocation of STAT1 homodimers, which bind to gamma-activated sequences (GAS) in the promoter of immunoregulatory genes.

IFN-stimulated genes are antiviral factors, positive regulators of the IFN pathway (STAT1 and 2 and IRF1) or negative regulators that help IFN-induced cells to return to cellular homeostasis (SOCS and UPS18) (6–11). Among antiviral genes, there are factors that function to increase cell sensitivity to PAMPs (OAS and PKR) or true antiviral effectors that block viral entry (Mx, IFITM, and TRIM), virus replication, translation and stability (IFIT, OAS, PKR, and ISG15), or viral release (viperin and tetherin/BST2) (8). While most IFN-induced factors known to date are proteins, IFN also activates the expression of several microRNAs that contribute to the antiviral state or to the control of IFN response (12). Few studies have been performed to address whether IFN could also regulate expression of long non-coding RNAs (lncRNAs) (13–15). In recent years, viral infection has been reported to be able to induce the expression of cellular lncRNAs. This has been shown for infection with enterovirus, influenza, HIV, hepatitis B and C viruses, and the SARS coronavirus (13, 16–23) (Carnero et al., in preparation). The lncRNA signature found after infection should be a mixture of transcripts induced by the virus and transcripts that respond to the cellular antiviral pathways activated by the infection. In fact, activation of TLRs by PAMPs induces the expression of several lncRNAs. TLR2 signaling leads to the activation of lncRNA-COX2, which regulates the expression of genes related to the immune system (24). Activation of TLR3 results in increased NEAT1, which increases the expression of genes such as IL8 (19). TLR4 controls IL1b-eRNA and IL1b-RBT46 lncRNAs whose downregulation diminishes IL1b and accumulation of LPS-induced RNAs (25). Likewise, the LPS-induced inflammatory response is controlled by lnc-IL7R (26). Innate activation also induces linc1992/THRIL, which controls TNFα and other genes involved in the immune response (27). In turn, TNFα induces Lethe, a pseudogene that responds to NFκB and reduces inflammation by inhibiting NFκB DNA binding activity (28). LncRNA responses are also critical for the functionality of dendritic cells, CD4+ and CD8+ T-cells (29–32). Thus, NEST lncRNA controls IFNγ locus in CD8+ T-cells leading to decreased *Salmonella enterica* pathogenesis (33, 34).

These studies illustrate the interest in identifying novel lncRNAs and elucidating their function and regulation. LncRNAs are thought to be at least as numerous as protein-coding genes, but only a few are well characterized (35–38). LncRNAs are transcripts similar to mRNAs but with poor coding potential. They are more cell type-specific, less expressed, and less well conserved than mRNAs (29, 39). Interestingly, lncRNAs are cell regulators that can function in *cis*, co-transcriptionally, or in *trans*. Some control the expression of coding genes located in the same genomic region. Therefore, the genomic location of lncRNAs can provide hints as to their functionality. They can be sense or antisense (when overlapping with one or more exons of another transcript in the same or in the opposite strand, respectively); intronic (when derived from an intron of another transcript); divergent or bidirectional (when they share a promoter with another transcript in the opposite strand and therefore are co-regulated); or intergenic (when they are independent, located in between two other genes). Several mechanisms are involved in the regulation of neighboring or antisense genes by lncRNAs. These include transcriptional activation or interference, recruitment of chromatin modifiers and remodelers, regulation of imprinting, editing, splicing or translation, and stability (40–44).

To address the issue of whether IFN could also regulate expression of lncRNAs, which may play key roles in the antiviral response, we analyzed the transcriptome of cells treated or not with IFNα2 by RNA sequencing (RNASeq). In this analysis, we identified two lncRNAs upregulated in response to IFN in different cell lines. Interestingly, these lncRNAs are expressed from positions in the genome divergent from the well-characterized ISGs ISG15 and BST2. Therefore, we have called them lncISG15 and lncBST2. These lncRNAs and their coding counterparts are also induced in cells infected with mutants of influenza or vesicular stomatitis viruses (VSV) that fail to block the IFN response. Surprisingly, they are also induced in culture cells infected with hepatitis C virus (HCV) and in the liver of patients with HCV infections. Finally, according to HUGO regulation, we have renamed lncBST2 BISPR, from BST2 IFN-stimulated positive regulator, as we show that inhibition of lncBST2 expression by RNAi leads to decreased levels of BST2 mRNA, providing a new layer of regulation of the IFN response.

## Materials and Methods

### Cells and patient samples

The HuH7 cell line, derived from a human hepatocarcinoma, was provided by Dr. Chisari’s lab (Scripps Research Institute, La Jolla, CA, USA). A549 cells, from human non-small cell lung carcinoma, were kindly provided by Estanislao Nistal (CIMA, University of Navarra, Spain). Human liver samples with or without HCV infection were obtained from the Biobank of the University of Navarra under approval from the Ethics and Scientific Committees. Liver tissue sections were snap frozen and stored at −80°C. The clinical data from HCV-infected subjects are shown in Table S1 in Supplementary Material.

### Cell culture

Cells were grown in Dulbecco’s Modified Eagle Medium (DMEM) supplemented with 10% fetal bovine serum (FBS) and 1% penicillin–streptavidin and maintained at 37°C in a 5% CO_2_ atmosphere. Twenty-four hours before treatment with IFN, HuH7 and A549 cells were seeded in six-well plates. Then, 0, 5, 50, 250, 1000, or 10000 units/ml of IFNα2 (Sicor Biotech, Lithuania) were used in a final volume of 2 ml. HuH7 cells were also treated with 250 ng/ml of IL28B/IFN-λ3 (R&D Systems) in a final volume of 2 ml. For treatment with ruxolitinib (Selleckchem), cells were seeded out 24 h before and treated with 0.8 μM ruxolitinib in a final volume of 2 ml. One hour after treatment media were discarded and replaced by media containing 100 units/ml IFNα. Cells were harvested for RNA extraction at the indicated times post-treatment.

### Cell transfections

siRNAs targeting lncBST2/BISPR were designed using iScore Designer and RNA Scales (45, 46) and purchased from Dharmacon. The lncBST2/BISPR siRNAs targeted the sequence GACUAGUGUGAGCAACAAA. For cell transfection with siRNAs, lipofectamine 2000 reagent (Invitrogen) was used according to manufacturer’s instructions. Cells were seeded 24 h before transfection. For each well of a six-well plate, 80 pmoles siRNA were used. The siRNA was mixed with 50 μl OPTIMEM. Furthermore, 6 μl lipofectamine were mixed with 250 μl OPTIMEM media and incubated for 5 min. Then, lipofectamine and siRNA solutions were mixed and incubated for 20–60 min at room temperature. After incubation, half of the volume of the cell media was discarded and 300, 150, or 75 μl of the lipofectamine mixture were added to each well of 6, 12, or 24-well plates, respectively. Six hours post-transfection the media from the cells was discarded and substituted with DMEM media enriched with 10% FBS and antibiotics.

### Virus infections

Hepatitis C virus JFH-1 was obtained from an initial viral stock from the genotype 2a JFH-1 plasmid (pJFH-1) previously described by Wakita et al. (47). The virus was amplified as described (15). Influenza virus strain A/PR8/34 WT (PR8), a mutant lacking NS1 (ΔNS1), VSV-GFP, and the mutant M51R were kindly provided by Estanislao Nistal (CIMA, University of Navarra, Spain) (48–50), Semliki Forest Virus (SFV) was a gift from Cristian Smerdou (CIMA, University of Navarra, Spain), and Adenovirus serotype 5 (Ad5) was amplified as previously described (48). VSV-eGFP titration was performed in quadruplicates on A549 cells. The supernatant from infected cells was collected and 1:10 serial dilutions were performed. Cells were seeded 24 h before infection in 96-well plates and infected with 50 μl of each dilution. Twenty-four hours after infection, GFP expression was visualized by microscopy and used to determine the titer. Cells were infected with HCV at a multiplicity of infection (moi) of 0.3, with VSV at a moi of 5 and with a moi of 10 of Influenza A, ΔNS1, Ad5, and SFV. In the case of the lytic viruses, we used a moi of 5 or 10 as this causes cytopathic effects at 24 h (for VSV, influenza and SFV) or 48 h (for Ad) in HuH7 or A549 cells. After infection, the virus was removed and fresh medium was added to the cells. Cells were harvested for RNA extraction at the indicated times post-infection.

### Cellular fractionation

Two million HuH7 cells were incubated in 100 μl of cytoplasmic buffer (50 mM Tris HCl pH7.4, 1 mM EDTA, and 1% NP40) for 5 min at 4°C. Then, cells were centrifuged for 5 min at 3000 *g* and the supernatant was used to isolate cytoplasmic RNA. The pellet was washed with cytoplasmic buffer and centrifuged as before. The supernatant was discarded and the pellet was used to isolate the nuclear RNA. RNA from nuclear and cytoplasmic fractions was isolated with MaxWell 16 research system (Promega).

### RNA extraction and quantitative RT-PCR

Human tissue was homogenized using the ULTRA-TURRAX dispersing machine (t25 basic IKA-WERKE) (51). Total RNA from the tissue was extracted in 1 ml TRIZOL (Sigma-Aldrich) and recommendations of the supplier were followed (52). DNase (Fermentas) treatment was performed to eliminate DNA from the samples before RT-PCR reactions. RNA was extracted from cells with the MaxWell 16 research system from Promega following the manufacturer’s recommendations. RNA concentration was measured using NanoDrop 1000 Spectrophotometer. The quality of the RNA was analyzed by Bioanalyzer (Agilent Technologies). Reverse Transcription (RT) was performed as described (53). The reaction was performed in the C1000 Touch Thermal Cycler from Bio-Rad. The samples were incubated at 37°C for 60 min, then at 95°C for 60 s and then immediately cooled to 4°C. qPCR was performed in the CFX96 Real-Time system from Bio-Rad as described (54). The results were analyzed with Bio-Rad CFX-manager software. GAPDH levels were evaluated in all the cases as a reference. Only the samples with similar GAPDH amplification were analyzed further. The primers used are listed in Table S2 in Supplementary Material and were designed with the Primer3 program^1^.

### High throughput sequencing

RNA of excellent quality, as determined by Bioanalyzer (Agilent Technologies) was treated with the Ribo-Zero rRNA removal kit (Epicenter) to deplete from ribosomal RNA. Library preparation with TruSeq RNA sample preparation kit (Illumina) and sequencing was performed at the EMBL genomics core facility (Genecore) in an Illumina HiSeq 2000. Sequences were paired-end, 150 bases long, and strand specific. RNASeq data are available at the NCBI Gene Expression Omnibus (GEO) data repository^2^.

### Bioinformatic analysis

RNA sequencing data analysis was performed using the following workflow: (1) the quality of the samples was verified using FastQC software; (2) the preprocessing of reads was performed by elimination of contaminant adapter substrings with Scythe and by quality-based trimming using Sickle; (3) the alignment of reads to the human genome (hg19) was performed using the Tophat2 mapper (55); (4) transcript assembly and quantification using FPKM of genes and transcripts was carried out with Cufflinks 2 (56); (5) the annotation of the gene locus obtained was performed using Cuffmerge with Gencode v16 as reference; and (6) differential expression analysis was performed using Cuffdiff 2 (56). Genes were selected as differentially expressed using a *p*-value threshold of 0.01. Further analysis and graphical representations were performed using an R/Bioconductor (57). Reads from all the differentially expressed sequences were visualized in the Integrative Genomics Viewer (IGV)^3^ (58, 59) and the sequences were compared to the ENSEMBL and ENCODE databases and searched for in the Genome Browser from UCSC^4^ for more information (60, 61). Candidates were divided into coding, non-coding (according to UCSC classification), or non-assigned, when the transcription of the sequence had not been annotated in the databases. Functional enrichment analysis of Gene Ontology (GO) categories was carried out using a standard hypergeometric test (62). Biological knowledge extraction was complemented through the use of Ingenuity Pathway Analysis (Ingenuity Systems)^5^, with a database that includes manually curated and fully traceable data derived from literature sources.

Open reading frame Finder (NCBI) was used to evaluate the length of all probable open reading frames (ORFs) in lncISG15 and lncBST2/BISPR. Coding potential was assayed with the coding potential assessment tool (CPAT) (63, 64) and by searching the LNCipedia database (65) for the presence of our candidates in the Pride archive (66) or in lists of transcripts associated with ribosomes (67, 68). Phylogenetic Codon Substitution Frequencies (PhyloCSF) were also used to predict the coding potential of lncISG15 and lncBST2/BISPR (69).

### Statistical analysis

Statistical analysis of the RNASeq data has been already described. Remaining analysis was performed using graph-path. Statistical significance of infected versus non-infected samples was calculated using a two-tailed non-parametric Mann–Whitney *t*-test or with a two-tailed Students *t*-test when the samples followed a normal distribution according to the Shapiro–Wilk test. Welch’s correction was applied for samples with heterogeneous variance. For correlation studies, a two-tailed non-parametric Spearman analysis was used. *P* values lower than 0.05 were deemed as significant.

## Results

### Identification of IFNα-regulated LncRNAs by RNASeq

To identify lncRNAs that respond to IFN, we treated HuH7 cells with 10000 units/ml of IFNα2 for 3 days. These conditions serve to induce the expression of well-known ISGs such as GBP1, IRF1, BST2, OAS, or ISG15 (15). In addition, this treatment induces an antiviral effect, as HCV-infected HuH7 cells treated with 10000 units/ml of IFNα2, show decreased levels of viral proteins and viral genomes compared to untreated infected cells (data not shown). Finally, the RNA isolated from HuH7 cells treated with 10000 units/ml of IFNα2 for 3 days was used to hybridize an Agilent array. Analysis of the array showed that well-characterized ISGs such as Mx1, STAT1, IRF9, ISG15, BST2, and several members of the GBP, OAS, and IFI families were upregulated with a very high statistical significance (*B* > 7) (15). Ingenuity analysis of the data showed that IFN signaling was the pathway with the highest enrichment followed by other antiviral responses.

**Figure 1:**
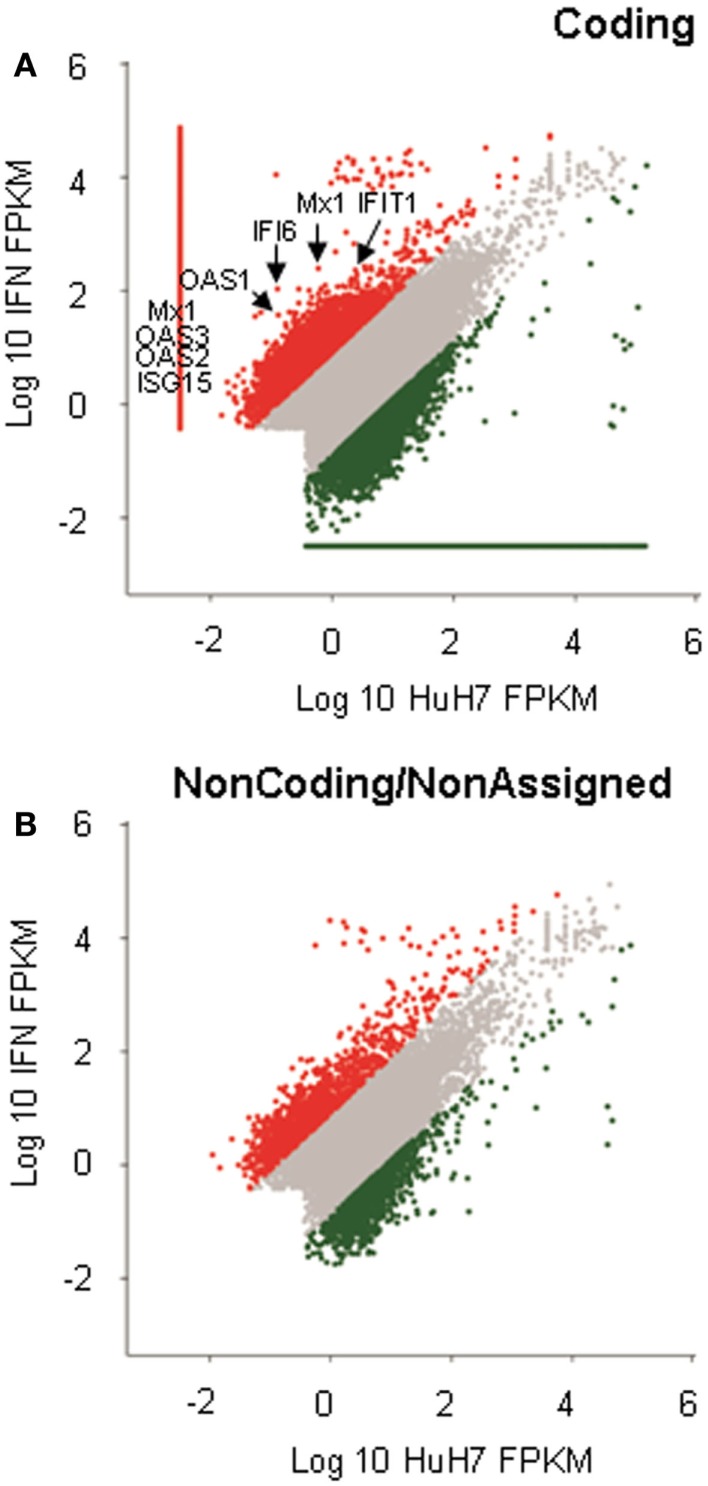
**RNASeq analysis of IFN-induced genes**. RNA isolated from HuH7 cells treated for 72 h with 0 or 10000 units/ml of IFNα2, was sequenced in an Illumina platform. More than 130 million reads per sample were obtained. Analysis of the sequences resulted in more than 200000 cufflink structures that were divided into coding **(A)** or non-coding/non-assigned **(B)** and positioned in dispersion graphs according to expression levels. Red dots show cufflink structures significantly upregulated in IFN-treated samples. Green dots are downregulated structures. The position of well-known ISGs is indicated **(A)**.

The microarrays were used to identify lncRNAs regulated by IFNα (15). However, an array will only evaluate the expression levels of the transcripts that hybridize to probes spotted in the array. In the case of the lncRNAs, the array used only addresses the expression of 7419 regions described as long intergenic non-coding RNAs (lincRNAs). However, it has been estimated that there could be as many lncRNA genes as coding genes, and some authors consider that the number of lncRNAs could be as high as ~200000 (37, 38). Therefore, to achieve a more complete identification of lncRNAs that respond to IFN, we analyzed the transcriptome by RNASeq. RNA isolated from control cells or HuH7 cells treated with IFN as described, was sequenced after ribodepletion. Around 130 million reads were obtained per sample. Analysis was performed using a bioinformatic workflow that includes Tophat2 and Cufflinks 2 as described in the methods section. The analysis showed that, among the genes upregulated in response to IFN, there were several ISGs such as Mx1, ISG15, BST2, or members of the IFI and OAS families (Figure 1A and Table S3 in Supplementary Material). Ingenuity analysis showed that IFN signaling is a top canonical pathway (*p* = 3.3 × 10^−3^), the top upstream regulator is IFNα2 (*p* = 1.9 × 10^−8^), and cell signaling and infectious and inflammatory diseases are among the main functions. The expression of ~1000 coding genes was altered by IFN (Table S3 in Supplementary Material).

The RNASeq analysis also showed that the expression levels of many regions that do not correspond to coding genes were also significantly modified in response to IFN (Figure 1B). Out of the 890 putative non-coding genes whose expression was significantly altered, half were upregulated (Table S3 in Supplementary Material). All candidates where visualized using IGV (Figure S1 in Supplementary Material) (58, 59). We also paid special attention to altered sequences located close to well-known ISGs and to genomic regions that were highly expressed and deregulated in response to IFN. Eight candidates that fulfill at least one of these two criteria were chosen for further validation (Table 1 and Figure S1 in Supplementary Material).

**Table 1:** **Characteristics of the lncRNA candidates**.

	Chr	Position	Length	UCSC	Ensembl	Validation	Ct	Rep Seq	Features
1	11	85195002–85195217F	216	Intron DLG2	ENSG00000150672	3.6 ×	20–21	Yes	LSU ribosomal RNA
2	13	110076414–110076761FR	348	No	None	3.8 ×	8–10	No	99% homology mitochondrial DNA
3	1	237766286–237766644R	359	miRNA inside	ENSG00000198626	5.0 ×	5–8	Yes	LSU ribosomal RNA
				Intron RYR2					
4	13	82264067–82264606F	539		ENSG00000214182	1.9 ×	29–31	Yes	
5	11	77597481–77597691R	211	Intron C11orf67 = AAMDC	ENSG00000087884	6.9 ×	5–7	Yes	
				Intron INTSA	ENSG00000149262				
6	9	79186718–79186900R	183		ENSG00000241781	3.7 ×	12–14	No	LSU ribosomal RNA
7	1	947220–948350	1130	Annotated	ENSG00000224969	21.1 ×	28–31	No	Close to ISG15
8	19	17516503–17529713F	13210	Annotated	ENSG00000269640	6530 ×	22–31	No	Close to BST2

*The table indicates for each candidate the chromosome and genomic position, the length of the peaks identified by the sequencing analysis, relevant UCSC information, Ensemble gene name, fold-change after IFN-treatment (validation), number of PCR cycles required for detection (Ct), presence of repetitive sequences (rep seq), and other special features*.

### IFN induces the expression of several LncRNAs

**Figure 2:**
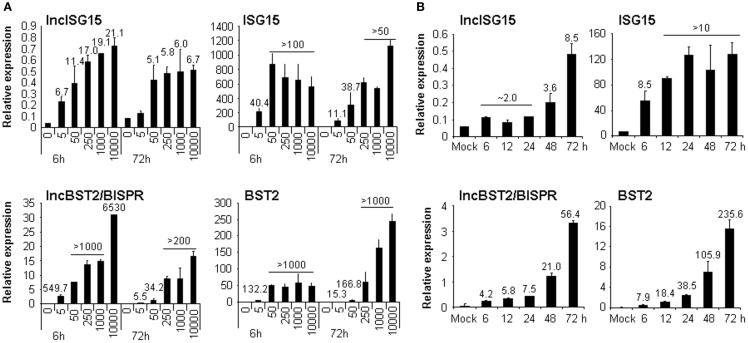
**lncISG15 and lncBST2/BISPR are induced by IFN**. HuH7 cells were treated with 0, 5, 50, 250, 1000, or 10000 units/ml IFNα2 **(A)** or with 250 ng/ml IFNλ for the indicated times **(B)**. Then RNA was isolated from each condition and used to evaluate the expression of lncISG15, lncBST2/BISPR, ISG15, BST2, and GAPDH mRNAs by qRT-PCR. GAPDH was used as a reference. The experiments were performed three times and each value shows the average of three replicas from a representative experiment. Error bars indicate standard deviations. When significant, the fold-change of treated versus non-treated cells is indicated at the top of each bar.

To validate the eight candidates chosen, we treated HuH7 cells with different doses of IFNα2. RNA was isolated from the cells at 6, 24, 48, or 72 h post-treatment and the expression levels of the candidates were evaluated by quantitative RT-PCR (qRT-PCR) (Table 1; Figure 2). All the candidates were induced after IFN-treatment from 2 to more than 1000-fold. However, many of the candidates were detected at very low cycles in the PCR amplification. A closer examination of their sequences indicated that they contained repetitive sequences or sequences similar to mitochondrial or ribosomal RNAs that could have led to an erroneous alignment of the RNASeq reads to the human genome. We believe that, even when the oligonucleotides used for amplification were specific, a partial homology to other sequences could allow cross-amplification and thus increased possibilities of misleading results. These candidates were not studied further.

We focused on two lncRNAs with no repetitive sequences whose expression was highly upregulated in response to IFN (Table 1; Figure 2). Interestingly, database analysis showed that they are expressed from positions in the genome located close to ISG15 and BST2, both of which are well-characterized ISGs. This may have functional relevance as some lncRNAs have been described to regulate the expression of neighboring genes. Therefore, we originally named these lncRNAs after their neighbor, lncISG15 and lncBST2. Later, lncBST2 was renamed BISPR to follow HUGO regulations. When we evaluated the expression of these lncRNAs and their neighboring transcripts, we observed that both were strongly upregulated at early times in response to IFN (Figure 2A). Furthermore, they responded to IFNα2 doses as low as 5 units/ml. These are similar levels to those found in the sera of some HCV patients (70). The induction was also observed at late times post-IFN-treatment. To evaluate further the robustness of the effect of IFN on these lncRNAs, we tested whether they also respond to IFNλ, a type III IFN. HuH7 cells treated with IFNλ for 6, 12, 24, 48, or 72 h also showed increased levels of lncISG15, lncBST2/BISPR, and their neighbors (Figure 2B). In this case, all the transcripts showed a higher upregulation at later times post-IFNλ treatment.

### Viral infections induce the expression of lncISG15 and lncBST2/BISPR

**Figure 3:**
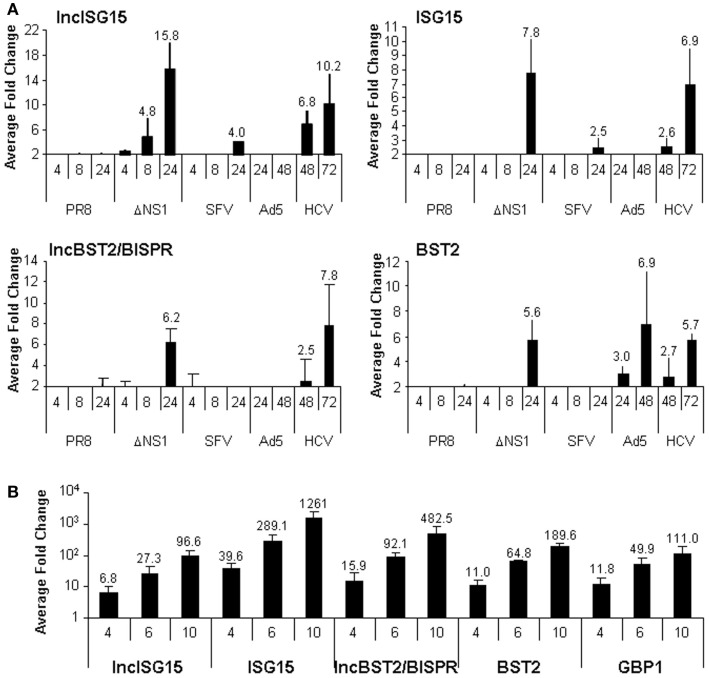
**LncISG15 and lncBST2/BISPR respond to viral infections in cultured cells**. **(A)** HuH7 cells were mock-treated or infected with wild-type influenza virus (PR8) or a mutant that lacks NS1 (ΔNS1), SFV, Ad5, or HCV for the indicated times. **(B)** A549 cells were mock-infected or infected with VSVM51R for 4, 6, or 10 h. Then, RNA was isolated and the expression levels of lncISG15, lncBST2/BISPR, ISG15, BST2 (**A** and **B**), GBP1 **(B)**, and GAPDH mRNAs were evaluated by qRT-PCR. GAPDH expression was used as a reference. The experiment was performed three times. Each value shows the average of three replicas from a representative experiment. Fold-changes of infected versus non-infected cells higher than two are indicated. Error bars indicate standard deviations.

Viruses activate the IFN response by several mechanisms. Therefore, they have evolved to block IFN production and the activation of the IFN pathway. The molecular mechanisms involved in this IFN blockade have been characterized for many viruses. Thus, for instance, NS1 protein from influenza virus and matrix protein from VSV are key factors in controlling IFN in infected cells (48–50, 71). We sought to check whether lncISG15 and lncBST2/BISPR were induced by the physiological IFN induced by an influenza virus that lacks NS1. Therefore, we evaluated the expression of these lncRNAs in cells infected with an influenza wild-type virus or a NS1 mutant. We also included cells infected with other RNA viruses such as SFV and HCV or DNA viruses such as adenovirus. All these viruses have developed mechanisms to block the cellular antiviral response and, with the exception of HCV, lead to a lytic infection. Different times post-infection were evaluated. The last point was collected when the cytopathic effect was apparent. This occurred at 24 h post-infection in the case of influenza and SFV or 48 h post-infection, in the case of adenovirus. HCV-infected cells were collected at 48 and 72 h post-infection. The results showed that at later times post-infection with the influenza virus lacking NS1, there was increased expression of lncISG15, lncBST2/BISPR, and their neighboring coding transcripts (Figure 3A). This increase was not observed in cells infected with wild-type influenza virus, or with other wild-type lytic viruses, suggesting that the induction may be mediated by IFN.

Most lncRNAs are tissue-specific. To determine whether lncISG15 and lncBST2/BISPR respond to infection only in HuH7 cells or whether this effect is specific for influenza viruses, we infected alveolar epithelial A549 cells with VSV-GFP wild-type virus or with a M51R matrix mutant that fails to control IFN. We chose A549, because lung cells serve as the primary site for productive infection of VSV and many respiratory viruses (72). Infection with the wild-type virus did not increase the expression of lncBST2/BISPR or BST2 (data not shown). However, A549 cells infected with the VSV mutant M51R for 4, 6, or 10 h did show increased levels of lncISG15, lncBST2/BISPR, ISG15, BST2, and other ISGs such as GBP1 (Figure 3B).

**Figure 4:**
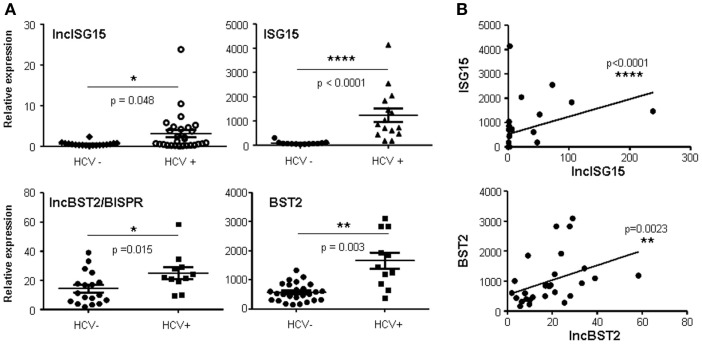
**LncISG15 and lncBST2/BISPR are increased in the liver of HCV-infected patients**. **(A)** Liver samples from HCV-negative and HCV-positive donors were used to quantify the levels of lncISG15, lncBST2/BISPR, ISG15, BST2, and GAPDH mRNAs. Statistical significance was calculated using a two-tailed non-parametric Mann–Whitney *t*-test for lncBST2/BISPR, ISG15, and lncISG15 and with a two-tailed Students *t*-test with Welch’s correction for BST2, which follows a normal distribution according to the Shapiro–Wilk test. **(B)** Expression levels observed for lncISG15, lncBST2/BISPR in patient samples were compared to the expression levels of their coding neighbors ISG15 and BST2, respectively. A correlation analysis was performed and statistical significance was calculated using a two-tailed non-parametric Spearman analysis.

Surprisingly, infection with HCV also increased the expression of lncISG15, lncBST2/BISPR, and other ISGs, including ISG15, BST2, and IRF1 (Figure 3A and data not shown). To determine whether these genes were also upregulated in infected patients, we used liver samples from HCV-negative and HCV-positive donors. After quantification of the RNA levels, we observed a significant increase in lncISG15, lncBST2/BISPR, ISG15, and BST2 in HCV-infected patients compared to controls (Figure 4A). With the number of patients evaluated, a significant correlation was not found between expression levels and infection with a particular genotype of HCV, presence of HCV-induced hepatocellular carcinoma (HCC), liver cirrhosis, or with a particular cirrhosis stage. Therefore, there were no significantly different levels of these transcripts in HCV-infected livers without HCC compared with the peritumoral tissue of HCV-infected livers with HCC. Although most of the samples belong to patients that are still alive, no significant correlation was observed between the levels of the evaluated transcripts and survival post-diagnosis. Finally, we performed correlation studies to analyze whether in the patients, the expression level of lncISG15 or lncBST2/BISPR correlates significantly with the expression level of their neighboring coding genes. The results show a highly significant positive correlation between lncISG15 and ISG15 or lncBST2/BISPR and BST2 (Figure 4B).

### Analysis of lncISG15 and lncBST2/BISPR promoters

The experiments performed so far suggest that a general correlation could exist between the expression of lncISG15 and ISG15 or lncBST2/BISPR and BST2. Each lncRNA and its neighboring coding gene have similar induction patterns in response to IFN or to viral infection (compare their levels in Figures 2 and 3). Furthermore, the levels of each coding/non-coding pair correlate significantly in patient samples (Figure 4B). To analyze this in more detail, we performed correlation studies of the coding/non-coding pairs in all the samples evaluated in Figures 2 and 3. The results show that the correlation of each pair was highly significant (Figure S2 in Supplementary Material). This suggests that they could be co-regulated, and therefore, they could share similar functions. However, expression of lncISG15 and lncBST2/BISPR also correlated significantly with the expression of other ISGs such as OAS, GBP1, or IRF1 (data not shown).

To obtain more information on the relationship between the coding/non-coding pairs, we searched several databases. LncISG15 and lncBST2/BISPR genes are in head-to-head orientation with their coding neighbors (Figure S3 in Supplementary Material) and they could share the same promoter. This is based on the following facts: (i) the distance between the two genes is <1000 bp, a cut-off for bidirectional promoters (73, 74); (ii) there is a single DNase hypersensitivity region located between the genes, and (iii) Polymerase II (Pol II) ChipSeq analysis of K562 cells shows a single peak covering the H3K27Ac region between both genes. Interestingly, the peaks observed for Pol II ChipSeq are increased at 30 min or 6 h post-treatment with IFNα or IFNγ. Finally, the promoter regions contain conserved ISRE sites and binding sequences for IRF1, IRF2, and IRF7.

**Figure 5:**
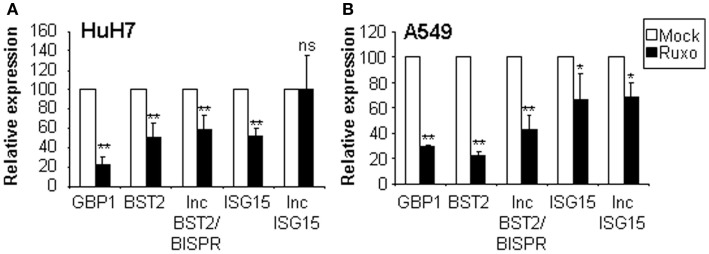
**Analysis of the expression of lncISG15, lncBST2/BISPR, and their coding neighboring genes after inhibition of the JAK/STAT pathway**. HuH7 **(A)** or A549 **(B)** cells were mock-treated or incubated with ruxolitinib for 1 h. Then IFNα was added for 8 h and RNA was isolated to quantify the expression levels of GBP1, BST2, lncBST2/BISPR, ISG15, lncISG15, and GAPDH, used as a reference. The experiment was performed twice in triplicates and the average value is indicated. Error bars indicate standard deviations. A star denotes statistical significance.

To discriminate whether lncISG15 and lncBST2/BISPR are induced directly by the JAK/STAT signaling pathway or by a secondary wave of the IFN response, we evaluated the expression of these lncRNAs and their coding neighboring genes in HuH7 or A549 cells incubated or not with the JAK/STAT inhibitor ruxolitinib. Expression of GBP1, a *bona fide* ISG, was also evaluated as a positive control (Figure 5). The results show that the levels of GBP1, BST2, and lncBST2/BISPR are significantly reduced in the presence of ruxolitinib, indicating that their expression is STAT-dependent. Levels of BST2 and lncBST2/BISPR were also reduced in cells treated with siRNAs targeting STAT1 or by inhibition of IRF1, a transcription factor that acts downstream of IFN (data not shown). This indicates that BST2 and lncBST2/BISPR respond to STATs but also to other transcription factors induced by IFN. These results agree with the possibility that BST2 and lncBST2/BISPR share a bidirectional promoter.

In contrast, the effect of the JAK/STAT pathway on ISG15 and lncISG15 expression was less robust. Treatment with ruxolitinib decreased the expression of ISG15 and lncISG15, but in the latter, this effect was only observed in A549 cells. No effect on ISG15 or lncISG15 expression was observed with a milder inhibition of STAT1 or inhibition of IRF1 using RNA interference (data not shown). Thus, although ISG15 is induced very rapidly after IFN-treatment, we do not observe a strong regulation of ISG15 or lncISG15 by the STAT pathway under the conditions used. In fact, it has been reported that a major regulator of ISG15 is IRF3, a transcription factor activated in response to PAMPs, but also a downstream effector of the IFN response (75).

### Analysis of the coding potential of lncISG15 and lncBST2/BISPR

**Figure 6:**
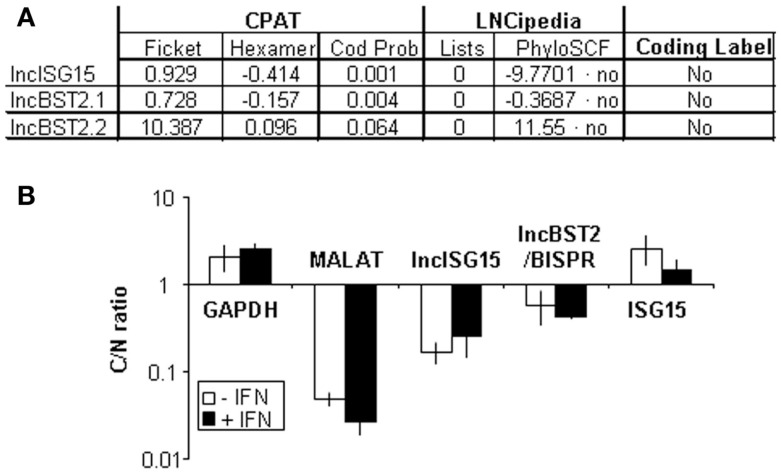
**lncISG15, lncBST2/BISPR have poor coding potential and accumulate preferentially in the nucleus**. **(A)** Bioinformatic analysis of the coding potential of lncISG15 and lncBST2/BISPR. Results obtained from CPAT and LNCipedia. Two transcripts have been evaluated for lncBST2/BISPR. lncISG15 and lncBST2/BISPR have a coding probability and a coding label of “non-coding RNAs” according to these analyses. “Lists” indicated the number of times that these transcripts have been found in the pride archive or in lists containing ribosome-associated RNAs published by Lee or Bazzini. See the text for other details. **(B)** Subcellular localization of lncISG15 and lncBST2/BISPR. HuH7 cells were mock-treated or treated with 10000 units/ml of IFNα2 and divided into nuclear and cytoplasmic fractions. RNA was isolated from each fraction and used to evaluate the expression levels of lncISG15 and lncBST2/BISPR by qRT-PCR. MALAT1, GAPDH, and ISG15 mRNA was also quantified and used as a reference to calculate the relative levels of each transcript and as a control to evaluate the subcellular fractionation. The ratio of cytoplasmic to nuclear levels is shown. The experiment was performed three times and each value shows the average of three replicas from a representative experiment. Error bars indicate standard deviations.

We evaluated the coding capacity of lncISG15 and lncBST2/BISPR bioinformatically. ORF Finder (NCBI) was used to determine all possible ORFs in these lncRNAs (Figure S4 in Supplementary Material). The analysis shows that all putative ORFs are shorter than 50 amino acids. Only two ORFs could be translated according to their poor susceptibility to nonsense mediated decay. However, these ORFs have non-consensus Kozak sequences at the initiation codon and therefore a poor coding capacity. Then, we evaluated the coding potential of lncISG15 and lncBST2/BISPR with the CPAT (63, 64) (Figure 6A). CPAT uses a model built with ORF size and coverage together with codon (Ficket score) and hexamer (hexamer score) usage bias. According to this program, lncISG15 and lncBST2/BISPR are non-coding as they have a coding probability of 0.001 and 0.064, respectively, much lower than 0.364, used as a threshold with the highest sensitivity and specificity to differentiate between coding and non-coding transcripts in humans (64). LncISG15 and lncBST2/BISPR were also described as non-coding in LNCipedia (65). This lncRNA database shows that these lncRNAs are not found in the Pride archive, a database for proteomic data, or in lists of transcripts associated with ribosomes in ribosome profiling experiments (66–68). Further, lncISG15 and lncBST2/BISPR were also described as non-coding by the analysis of PhyloCSF, which uses multiple alignments to calculate the phylogenetic conservation score and determines whether a multi-species nucleotide sequence alignment is likely to represent a protein-coding region (69).

Finally, we evaluated the subcellular localization of lncISG15 and lncBST2/BISPR in HuH7 cells mock-treated or treated with 10000 units/ml of IFNα. RNA was isolated from nuclear or cytoplasmic fractions and quantified by qRT-PCR. We found that the coding GAPDH or ISG15 mRNAs accumulate preferentially in the cytoplasm while the nuclear RNAs MALAT1 or U6 are preferentially nuclear (Figure 6B data not shown). Similarly, lncISG15 and lncBST2/BISPR, compared to mRNAs, accumulate preferentially in the nucleus. This result, together with the bioinformatic analyses, strongly suggests that lncISG15 and lncBST2/BISPR are non-coding RNAs.

### LncBST2/BISPR regulates the expression of BST2

**Figure 7:**
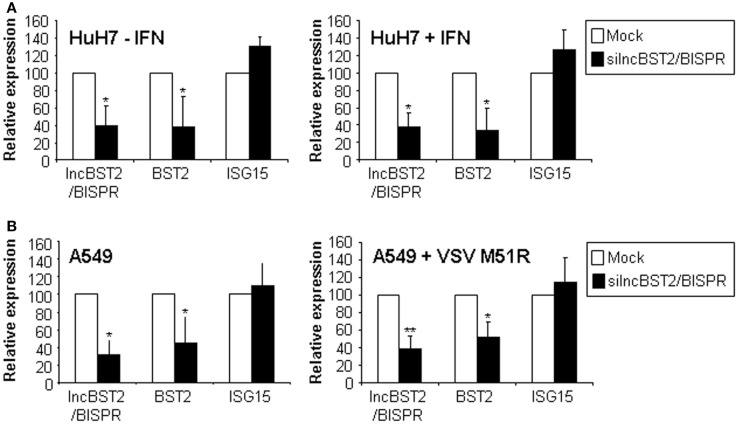
**LncBST2 controls the expression of BST2 mRNA**. HuH7 **(A)** or A549 **(B)** cells were transfected with control siRNAs (Mock) or siRNAs targeting lncBST2 for 48 h. HuH7, cells were treated or not with IFNα for 6 h before RNA isolation **(A)**. A549 cells were infected or not with VSV M51R **(B)**. Levels of lncBST2, BST2, ISG15, and GAPDH mRNAs were evaluated by qRT-PCR. The experiments were performed at least three times. Values are refereed to 100% of the control samples. Error bars indicate standard deviations from a minimum of three independent experiments.

To address the role of lncBST2/BISPR, we used RNA interference. HuH7 cells treated or not with IFN, were transfected with siRNAs targeting lncBST2/BISPR and RNA expression was evaluated by qRT-PCR. The results show that expression of lncBST2/BISPR was decreased compared to cells transfected with control siRNAs (Figure 7A). Surprisingly, inhibition of lncBST2/BISPR also led to decreased levels of BST2 mRNA. Expression of lncISG15, ISG15, GBP1 or expression of genes located in the genome close to BST2 or lncBST2/BISPR, such as GTPBP3 or MVB12A, was not affected (Figure 7A and data not shown). To determine whether this was a general phenomenon, we transfected the siRNAs targeting lncBST2/BISPR into A549 cells infected or not with the VSV M51R mutant or treated with IFN. Similarly to what has been observed in HuH7 cells, the siRNA that targets lncBST2/BISPR leads to decreased levels of lncBST2/BISPR and BST2 mRNA while the levels of ISG15 mRNA are not significantly affected (Figure 7B). Similar results were observed with a different siRNA targeting lncBST2/BISPR.

## Discussion

RNA sequencing analysis of human cells treated with IFNα2 and controls has allowed the identification of lncRNAs induced in response to IFN (Figure 1). Analysis of the RNASeq data shows that several of the upregulated genes are well-known coding ISGs such as ISG15 or OAS (Figure 1A, Table S3 in Supplementary Material). Ingenuity analysis confirms the enrichment of genes involved in the IFN response among the regulated factors. We have used RNAs from similar IFN-treated and control cells to hybridize expression arrays (15). Comparison of the datasets obtained in the analysis of the array and the RNASeq shows that only 13 coding genes were identified in both studies, including OAS, ISG15, Mx1, and some members of the IFI family. Generally, overlap between microarray and RNASeq analysis is not high (76). Furthermore, the overlapping decreases with sequencing depth and when low fold-changes or low abundance genes are analyzed. (77). This is because sequencing of low transcript abundances is characterized by high variance, which impedes their identification in RNASeq analysis. We believe that this may explain the poor correlation found between the array and the RNASeq datasets. In fact, we have determined by qRT-PCR that some IFN-related low abundance transcripts are detected only in the array analysis. These are early responders to IFN, which increased only marginally 3 days after IFN-treatment, when the analysis was performed. Therefore, we believe that some lncRNAs induced early post-IFN-treatment may have not been identified in our analysis. Interestingly, in the process of writing this manuscript, a paper was accepted describing the identification of IFN-induced lncRNAs by RNASeq in samples treated with IFN for short times (14). We believe that this study will be complementary to our work. Together, the datasets should contain lncRNAs regulated at early and later times post-IFN-treatment. Similarly, the lack of correlation between the microarray and RNAseq datasets also indicates that they can complement each other.

We have identified two lncRNAs whose expression is highly upregulated in response to different doses of IFNα (Table 1; Figure 2A) or IFNλ (Figure 2B). Our results show that induction of these lncRNAs by IFNα seems faster than that observed for IFNλ. We cannot rule out the possibility that a fast response to IFNλ may also be observed when higher doses are used. ENCODE analysis of polymerase II binding to the promoters of these lncRNAs also shows that they may be induced by treatment with IFNα and IFNγ (Figure S3 in Supplementary Material). These lncRNAs have been named lncISG15 and lncBST2/BISPR after their neighboring genes, which play a key role in the antiviral IFN response. Our molecular and bioinformatic analyses strongly suggest that lncISG15 and lncBST2/BISPR are indeed lncRNAs, as they accumulate preferentially in the nucleus of IFN-treated or untreated cells (Figure 6B) and they have poor coding potential (Figure 6A and Figure S4 in Supplementary Material).

In general, the upregulation of lncISG15 and lncBST2/BISPR mimics that of their coding counterparts (Figures 2–4). In fact, analysis performed with all the expression data obtained in our studies, shows a highly significant correlation between lncISG15 and ISG15 and between lncBST2/BISPR and BST2. Significant correlations also exist between these lncRNAs and other IFN-induced genes such as OAS, GBP1, or IRF1 (Figure S2 in Supplementary Material and data not shown). This may reflect the fact that all these genes are induced by IFN with a similar kinetics. In the case of the lncRNAs and their coding counterparts, correlation of the expression may result from their transcriptional co-regulation. Experimental and bioinformatic analyses indicate that BST2 and lncBST2/BISPR are *bona fide* ISGs strongly induced by the JAK/STAT pathway in response to IFN (Figure 5 and Figure S3 in Supplementary Material). Furthermore, expression of BST2, ISG15, and their neighboring non-coding genes is induced by downstream effectors of the IFN response. These studies allow us to suggest that lncISG15/ISG15 and lncBST2/BISPR/BST2 may share bidirectional promoters. Other IFN-induced gene pairs may also be co-regulated by bidirectional promoters as these are enriched in STAT1 binding (78).

Bidirectional promoters often couple genes involved in the same process, allowing for coordinated temporal and environmental responses (73, 78–82). Non-coding RNAs generated from bidirectional promoters may have functional roles that affect the bidirectional promoter, the neighboring protein-coding gene, or more distal genes (83). These effects could lead to activation or repression of the expression and could be mediated by either the transcription process itself or by the produced ncRNA transcript (84). In this study, we show that post-transcriptional inhibition of lncBST2/BISPR leads to reduced levels of BST2 mRNA (Figure 7). Therefore, lncBST2/BISPR should increase transcription or stability of its coding neighboring gene. Our results demonstrate that this regulation seems specific for BST2, as lncBST2/BISPR downregulation does not affect the expression of genes located nearby, which has been described for compact genomes (85). Moreover, inhibition of lncBST2/BISPR does not affect expression levels of other IFN-related genes such as ISG15 or GBP1 (Figure 7 and data not shown).

We anticipate that inhibition of lncBST2/BISPR, and therefore of BST2, could impact the antiviral effects of IFN. BST2 is also named tetherin, as it inhibits viral budding by using anchors that trap virions on the cell membrane (86–88). Several enveloped viruses have been shown to be susceptible to the action of BST2/tetherin and have evolved to develop evasion strategies (87). Interestingly, HIV, influenza, HCV, and VSV are among the susceptible viruses and could be used to test the antiviral role of lncBST2/BISPR (49, 89–95). In fact, we show that lncBST2/BISPR and BST2 are induced after infection with HCV or influenza and VSV mutant viruses that activate the IFN response (Figure 3).

Upregulation of lncBST2/BISPR, lncISG15, and their coding neighbors was also observed in patients infected with HCV compared to controls (Figure 4). Similarly, a significant upregulation of lncBST2/BISPR and ISG15 was also detected in human T-lymphocytes infected with HIV compared to controls (data not shown). A non-significant increase in BST2 and lncISG15 was also observed in these samples. This leads to the possibility that interference with these factors could have therapeutic relevance. It is unclear why cells infected by these viruses, which employ several viral proteins to block the IFN pathway, show activation of these IFN response genes (96). In the case of HCV, it has been previously shown that patients with chronic HCV infections express ISGs, including high levels of ISG15 (97–99). In fact, HCV has evolved to use some ISGs for viral replication (100, 101). This is the case for ISG15. ISG15 is an ubiquitin-like protein that attaches to its targets in a process called ISGylation (102, 103). Protein ISGylation may result in increased or decreased functionality depending on the target (104). ISG15 preferentially conjugates newly synthesized proteins affecting more strongly viral proteins or cellular proteins translated into IFN-induced cells (105). Viruses such as influenza, HIV, or VSV are susceptible to the action of ISG15 (103, 106). In the case of HCV, a pro-HCV role for ISG15 has been reported (105, 107). ISG15 has been shown to negatively regulate RIG-I and thus to inhibit the signaling process leading to IFN induction that affects HCV replication (108). Furthermore, ISG15 expression in the liver of chronically infected patients is considered a negative predictive biomarker of the ability of the patients to respond to IFN therapy (97–99) (Figure 4). In our study, we cannot address whether lncISG15, BST2, or lncBST2/BISPR are markers for the susceptibility of HCV patients to respond to IFN-treatment, as the HCV patients that we have studied are non-responders to IFN.

We believe that, similar to lncBST2/BISPR, lncISG15 could affect the expression of ISG15 or other genes. This lncRNA-mediated control has also been described for a lncRNA located close to the ISG viperin, which has been shown to regulate the levels of many IFN-inducible genes (14, 109). Further experiments will be required to address the role of lncISG15 and to decipher the molecular mechanisms that allow the control exerted by lncBST2/BISPR on BST2. We believe that these studies may be important to better understand the IFN response and its pro or antiviral functions on HCV and other viruses.

## Author Contributions

Marina Barriocanal and Elena Carnero designed and performed the experiments and analyzed and interpreted the data; Marina Barriocanal contributed to the writing of the manuscript; Victor Segura was in charge of all the bioinformatic analyses; and Puri Fortes conceived the project and the required experiments, provided the budget, interpreted the data, and wrote the manuscript.

## Conflict of Interest Statement

The authors declare that the research was conducted in the absence of any commercial or financial relationships that could be construed as a potential conflict of interest. The Review Editor Saba Valadkhan declares that, despite having collaborated on a publication in the last 2 years with authors Puri Fortes, Marina Barriocanal, and Elena Carnero, the review process was handled objectively.

## Supplementary Material

The Supplementary Material for this article can be found online at http://www.frontiersin.org/Journal/10.3389/fimmu.2014.00655/abstract

## Footnotes

^1^http://frodo.wi.mit.edu

^2^http://www.ncbi.nlm.nih.gov/geo/

^3^http://www.broadinstitute.org/igv

^4^http://genome.ucsc.edu

^5^http://www.ingenuity.com

## Author Note

A manuscript from Dr. Valadkhan’s laboratory has been accepted for publication that describes similar results (110).
