# Supplementary material for: Long Non-Coding RNA BST2/BISPR is Induced by IFN and Regulates the Expression of the Antiviral Factor Tetherin
Source: Front Immunol. 2015 Jan 9;5:655. doi: 10.3389/fimmu.2014.00655 (PMC4288319; doi:10.3389/fimmu.2014.00655)
Supplement: Supplementary file 1 [file Data_Sheet_1.ZIP › Captions.docx]

***Supplementary Material***

**Long non-coding RNA BST2/BISPR is induced by IFN and regulates the expression of the antiviral factor Tetherin**

**Marina Barriocanal^1^*, Elena Carnero****^1^*, Victor Segura^2^ and Puri Fortes^1$^**

1, 2. Center for Applied Medical Research (CIMA). Department of Gene therapy and Hepatology (1) and Bioinformatics unit (2). University of Navarra. Pamplona. Spain.

* These authors have contributed equally.

$ To whom correspondence should be addressed. CIMA. Pio XII 55. Pamplona 31008. Spain. Ph: 34948194700. Fax: 34948194717. E-mail: pfortes@unav.es

1. **Supplementary Figures and Tables**

**Table S1. Clinical data for the HCV-infected patients used in this study.** The table shows the age, gender and the date the sample was collected from a liver transplantation (tx) or a hepatectomy (hepa). The virus subtype is also indicated for most of the patients.

**Table S2. Sequences of the oligonucleotides used in this study.**

**Table S3. List of coding and non-coding genes identified as differentially expressed in the RNASeq analysis.** The table includes a gene ID, the gene short name (for coding) or the locus (for non-coding), the relative values or the fpkm for each sample, the log2 fold-change and the p value.

**Figure S1.** Maps of selected IFN-regulated non-coding RNAs are shown using Integrative Genomics Viewer (IGV).

**Figure S2. Correlation of expression between lncISG15, lncBST2**/BISPR**, and their coding partners.** Expression levels observed for lncISG15 and lncBST2**/**BISPR were correlated with those of their coding neighbors ISG15 and BST2, respectively. Statistical significance was calculated using a two-tailed non-parametric Spearman analysis.

**Figure S3. UCSC map of lncISG15, lncBST2/BISPR, and their coding partners.** UCSC representation of the genomic location of lncISG15 and ISG15 (A) and lncBST2**/**BISPR and BST2 (B). From top to bottom genomic positions, structure of each coding and non-coding gene arranged in exons and introns, location of CpG islands, peaks of H3K27Ac marks, location of conserved transcription factor binding sites and peaks of polymerase II according to Chip-Seq data of K562 control cells or cells treated with IFNα or IFNγ for 30 minutes or 6 hours extracted from ENCODE are shown.

**Figure S4. Analysis of potential ORFs expressed from lncISG15 and lncBST2/BISPR.** The analysis with ORF Finder (NCBI) shows all start (green) and stop (pink) codons in the three frames (left). The total length and the position of the exon-exon junctions is indicated with an arrowhead. The nucleotide position and the peptide length are indicated for each ORF longer than 35 aa (right). ORFs with poor susceptibility to NMD are shown in red and analyzed in more detail. Nucleotides that differ from the Kozak consensus sequence gccRccAUGG, where R is A or G, are indicated in red. The predicted isoelectric point (ppI) of the putative protein is also indicated.
