# Supplementary figures and images for: Long Non-Coding RNA BST2/BISPR is Induced by IFN and Regulates the Expression of the Antiviral Factor Tetherin
Source: Front Immunol. 2015 Jan 9;5:655. doi: 10.3389/fimmu.2014.00655 (PMC4288319; doi:10.3389/fimmu.2014.00655)

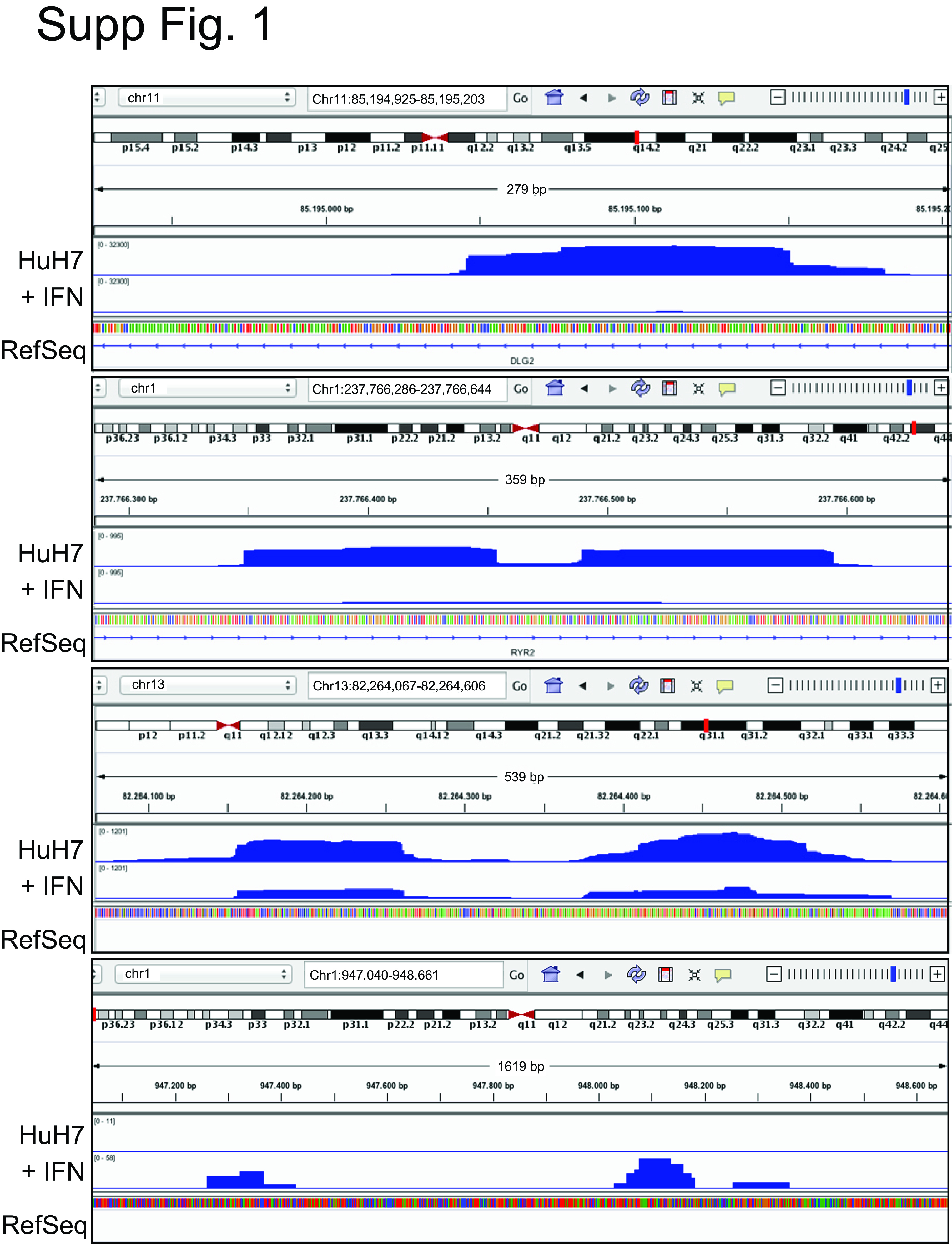

Supplement: Supplementary file 1 [file Data_Sheet_1.ZIP › Figure S1.jpg]

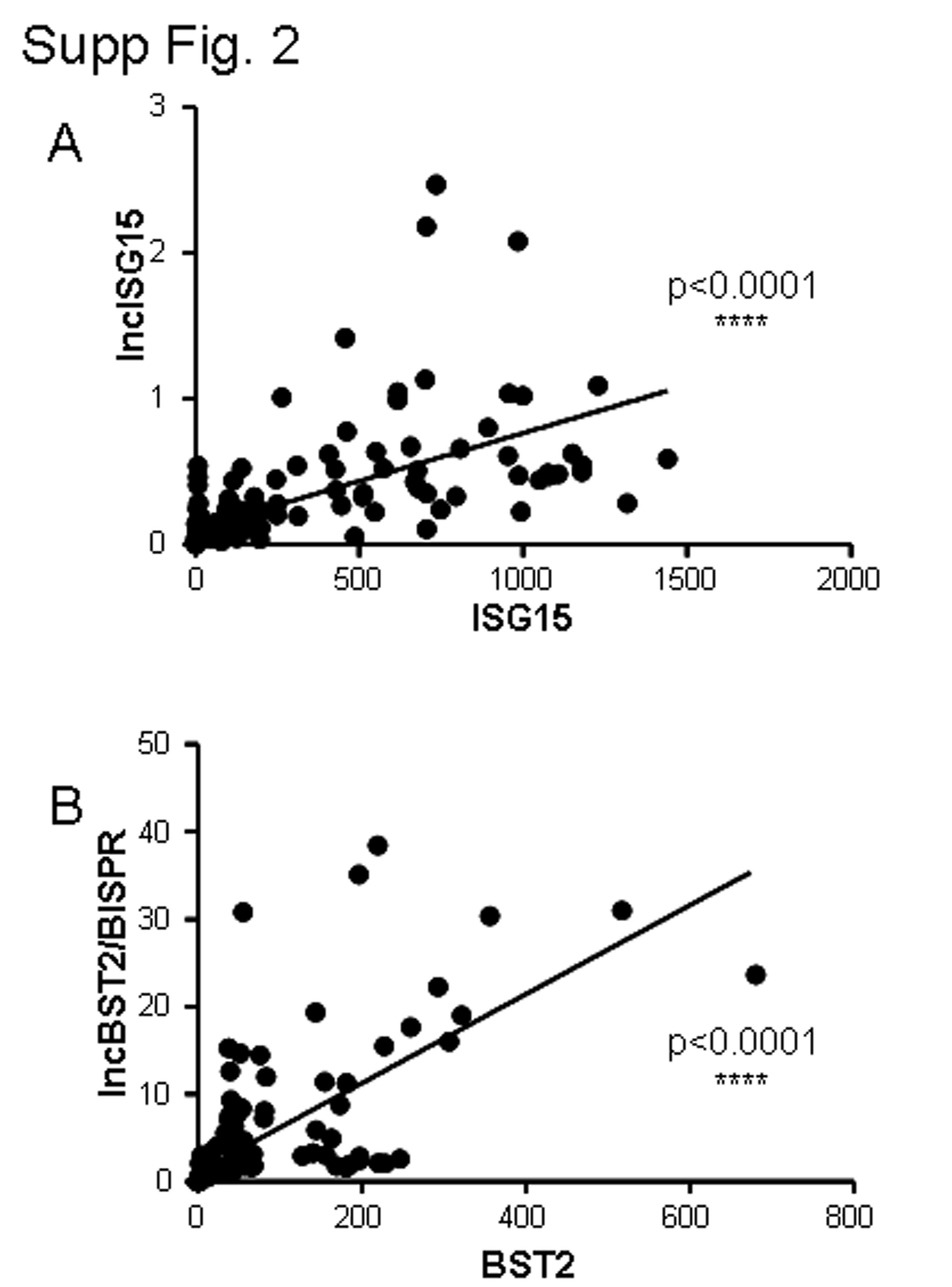

Supplement: Supplementary file 1 [file Data_Sheet_1.ZIP › Figure S2.jpg]

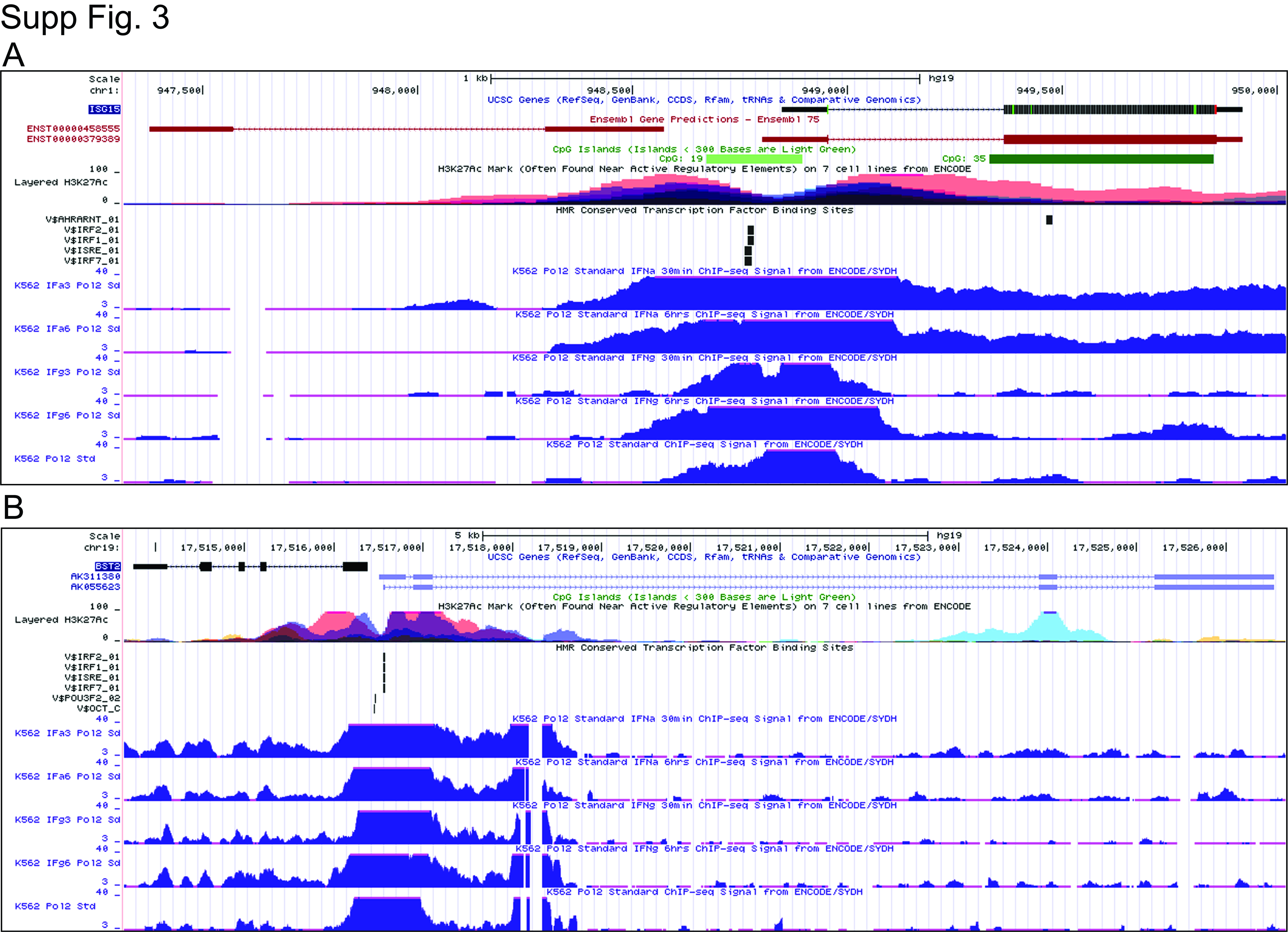

Supplement: Supplementary file 1 [file Data_Sheet_1.ZIP › Figure S3.jpg]

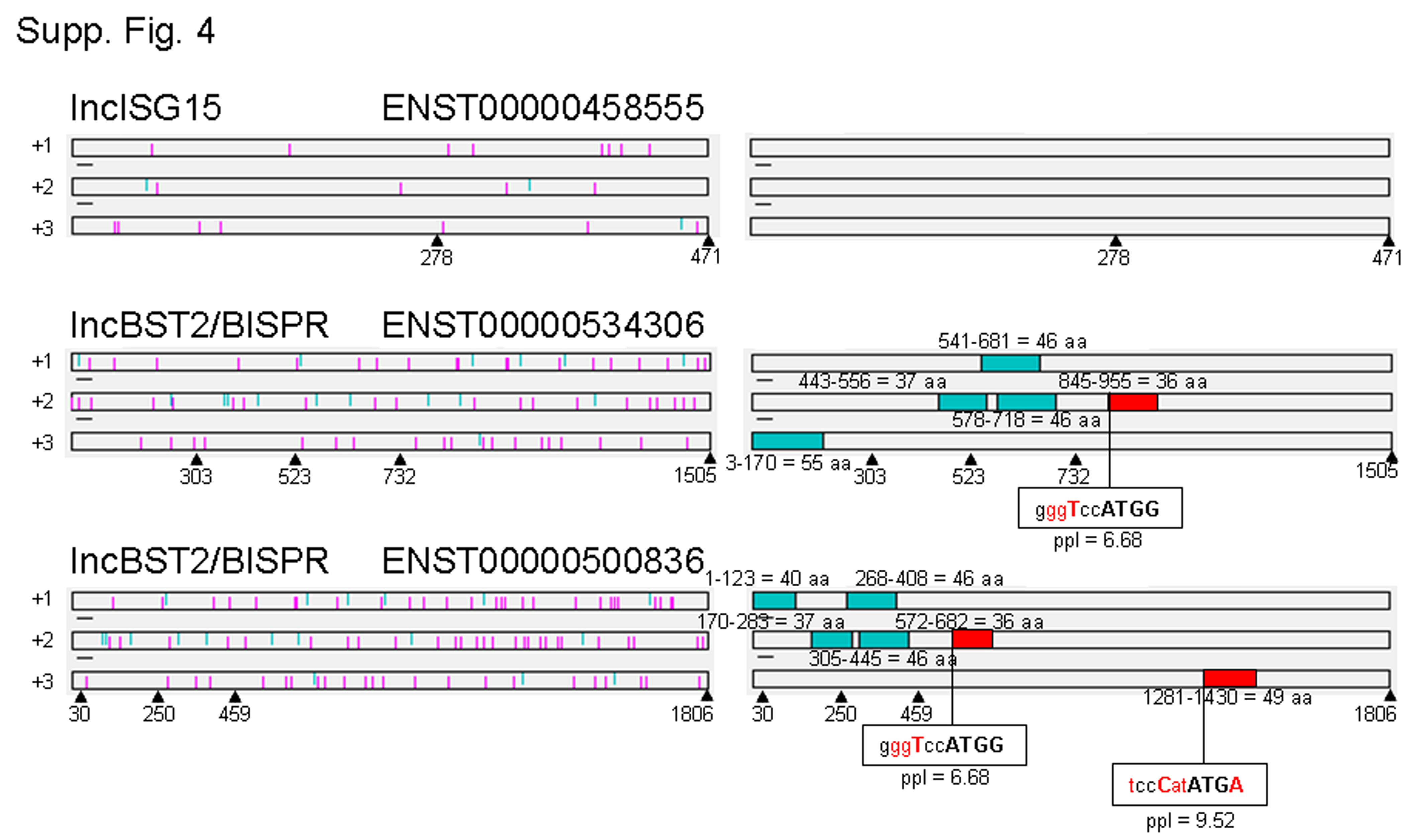

Supplement: Supplementary file 1 [file Data_Sheet_1.ZIP › Figure S4.jpg]
